# Air Pollution, Oxidative Stress, and Alzheimer's Disease

**DOI:** 10.1155/2012/472751

**Published:** 2012-03-15

**Authors:** Paula Valencia Moulton, Wei Yang

**Affiliations:** ^1^The Environmental Sciences and Health Graduate Program, University of Nevada, Reno, NV 89557-0186, USA; ^2^School of Community Health Sciences, University of Nevada, Reno, NV 89557-0274, USA

## Abstract

Alzheimer's disease (AD) is the most common form of dementia affecting millions of people worldwide and will continue to affect millions more with population aging on the rise. AD causality is multifactorial. Known causal factors include genetic predisposition, age, and sex. Environmental toxins such as air pollution (AP) have also been implicated in AD causation. Exposure to AP can lead to chronic oxidative stress (OS), which is involved in the pathogenesis of AD. Whereas AP plays a role in AD pathology, the epidemiological evidence for this association is limited. Given the significant prevalence of AP exposure combined with increased population aging, epidemiological evidence for this link is important to consider. In this paper, we examine the existing evidence supporting the relationship between AP, OS, and AD and provide recommendations for future research on the population level, which will provide evidence in support of public health interventions.

## 1. Introduction

Air pollution is a well-known environmental hazard and its association with respiratory and cardiovascular pathology has been consistently confirmed by several epidemiological studies. In recent years, the idea that air pollution might be also associated with Alzheimer's disease (AD) and other neurodegenerative disorders has been the focus of many toxicological studies. However, the epidemiological evidence supporting this association is limited.

Alzheimer's disease—a progressive and irreversible neurodegenerative disorder—is the most common form of dementia among older adults affecting more than 4 million people in the USA and almost 30 million worldwide [1–3]. The most proven risk factor for AD is advanced age. Other common risk factors include a positive family history of AD [1], the presence of APOE-4 alleles [4, 5], being a member of the female sex [6], cardiovascular disease [7], head injury [8], Down syndrome [1, 9], and low educational level [10]. As people continue to live longer, it is likely that the number of Alzheimer's cases will increase. By 2050, it is projected that approximately 13 million people in the USA and 100 million people worldwide will be affected by this disease [2, 11]

Increasing evidence indicates AD and other neurodegenerative disorders are at least partially mediated by oxidative stress. Oxidative stress is the state of redox imbalance that results from a production of reactive oxygen species (ROS) that exceeds the capacity of antioxidant defense mechanisms [12]. Environmental exposures such as air pollution can enhance an organism generation of ROS; thus, air pollution exposure could very well represent a risk factor for AD by enhancing oxidative stress processes capable of inducing physiological alterations of the central nervous system.

Air pollution is a prevalent environmental hazard. In the USA, National Ambient Air Quality Standards (NAAQSs) have been established for six principal air pollutants—criteria air pollutants—proved to represent a threat for human health. These pollutants include (1) ozone (O_3_), (2) particulate matter (PM), (3) carbon monoxide (CO), (4) nitrogen oxides (NO_x_), (5) sulfur dioxide (SO_2_), and (6) lead. In spite of the current standards, it is estimated that in the USA over one hundred million people live in areas that exceed the recommended air quality levels [13].

The large number of individuals exposed to air pollution levels above the recommended standards and population aging are two factors that could act synergistically to increase the prevalence of AD. Even after accounting for the predicted increase in Alzheimer's frequency due to population aging, the significant prevalence of air pollution could very well exacerbate the impact of this disease on public health. Granting that air pollution could be one of the factors involved in AD causality, its widespread occurrence makes ascertaining its association with AD a public health priority. The association between air pollution—specifically PM and O_3_—and AD via oxidative stress is the focus of this paper.

## 2. Aging, Oxidative Stress, and Alzheimer's Disease

### 2.1. Oxidative Stress

 Reactive oxygen species (ROS) is a term used to collectively refer to “reactive” molecules containing oxygen, which include free radicals and derivates, and are capable of leading to oxidative changes within cells [14–16]. A wide variety of ROS are produced in healthy tissues in the course of normal metabolism at different cellular sites. However, the main source of ROS is the mitochondrial electron transport chain, specifically complex I (NADH dehydrogenase) and III (ubiquinone-cytochrome *c* reductase) of the chain [16–21]. Other important generators of ROS in vivo include peroxisomal fatty acids metabolism, cytochrome P-450 reactions, phagocytic cells (respiratory burst), and numerous enzymes [19, 22]. ROS are important for maintaining oxygen homeostasis in tissues and destructing microbial invaders [15]. However, they can also cause oxidative changes within the cell [16] and modify proteins, lipids, and nucleic acids to develop or enhance age-related manifestations [12, 21, 23].

Several antioxidant systems—enzymes, vitamins, and metabolites—protect the cell against ROS-mediated oxidative damage by three key mechanisms: (1) scavenging ROS and their precursors, (2) binding catalytic metals ions used for ROS formation, and (3) generating and upregulating endogenous defense mechanisms [12, 23–27]. The balance between ROS production and antioxidant defense system determines the degree of oxidative stress [17]. When ROS formation exceeds the capacity of the antioxidant defense systems oxidative stress occurs, which results in oxidative damage to macromolecules—lipids, proteins, and nucleic acids—mitochondria, and other cells compartments [12, 16, 19, 22, 24, 25, 27, 28].  Table 1 lists some examples of ROS, ROS sources, and antioxidant defense mechanisms [17–19, 22, 26]. 

### 2.2. Oxidative Stress and Aging

From a biological perspective, aging is defined as the accumulation of changes over time responsible for the chronological alterations that occur with age and result in an increased risk of disease and death with advanced age [28, 29]. No theory has been widely accepted to explain the aging process [28]. However, the oxidative stress hypothesis proposed by Denham Harman in 1956—the Free Radical Theory of Aging—offers the best mechanistic explanation of aging and age-related diseases [28, 29]. Harman's theory posits a single common process modified by genetic and environmental factors that is responsible for aging and subsequent death. Harman argues aging is primarily the result cumulative damage on macromolecules resulting from free radical attacks—oxidative stress [28, 30, 31]. This theory was later expanded to propose mitochondria as the main source of free radical production [28, 31]. Consequently, aging can now be described as the result of the accumulation of ROS-induced damage to macromolecules by free radicals mainly of mitochondrial origin [19, 28].

In addition to being a main source, mitochondria are also a major target of ROS. Mitochondrial DNA is especially vulnerable to oxidative damage because of its proximity to the site of mitochondrial ROS production (mitochondria), little protection of its structure (no full histone coat), and limited repair mechanisms [17, 19–21, 29, 32]. During normal aging, free radical damage to mitochondria and ROS production increase and antioxidant mechanisms become progressively impaired [28, 33]. Consequently, vulnerability to oxidative stress and accumulation of oxidatively damaged macromolecules increase with age and may contribute to aging and age-related degeneration [20, 28].

Although fast replicating cells with low levels of oxygen consumption do not suffer free radical damage, mitochondria of highly differentiated cells with high levels of oxygen utilization are highly vulnerable to oxidative stress [29]. Consequently, neurons are especially vulnerable to mitochondrial damage by free radicals [29, 34]. Thus, the detrimental effects of the aging process are best observed in the brain, where irreversibly damaged cells cannot be replaced [21].

### 2.3. Oxidative Stress and Alzheimer's Disease

The brain—an organ rich in fatty acids, consumer of high levels of energy and physiological oxygen, and poor in antioxidant defense mechanisms—is particularly vulnerable to oxidative stress [16, 23, 27, 34–38]. Depending on the macromolecule targeted by ROS, oxidative stress will manifest as lipid peroxidation, protein oxidation, or DNA oxidation. Accumulation of oxidation of lipids, proteins, and DNA by free radicals are responsible for the functional decline in aged brains, which manifests as a deterioration in cognitive function and motor skills [12, 19].

Alzheimer's disease is a progressive and irreversible neurodegenerative disease manifested as a slowly progressive dementia, which begins with subtle memory loss and progresses to severe decline in cognitive function and disability [1, 39, 40]. The neuropathological hallmarks of AD are senile plaques and neurofibrillary tangles [41]. Evidence of an increased oxidation of macromolecules—lipids, carbohydrates, proteins, and DNA—and oxidative stress products has been found in senile plaques and neurofibrillary tangles [25]. Biomarkers of these forms of oxidation have been observed not only in AD brains but also in peripheral tissues (e.g., blood cells) and biological fluids (e.g., urine) of individuals affected by AD [16, 23, 37, 38, 42–44].

The principal component of senile plaques found in Alzheimer's brains is amyloid-beta (A*β*) peptide [45, 46], which plays an important role in the etiology and progression of this disease [45]. However, it is unclear whether A*β* peptide deposition is the cause or the result of the oxidative stress observed in Alzheimer's brains [27, 46]. In the initial phase of the development the disease, A*β* peptide deposition and the formation of neurofibrillary tangles are consequences of oxidative stress and may serve as shields to protect neurons against oxidative damage. However, as the disease progresses, these evolve into prooxidants driving a self-sustained “autodestructive” process and the progression of the disease [16, 47].

Amyloid-beta peptide-related oxidative stress provides a theoretical framework that unites these two components in the pathogenesis of AD [46] and suggests two possible scenarios: first, neuronal degeneration could be the result of an oxidative stress response to senile plaques and neurofibrillary tangles rather than to these lesions as such [47]. Second, oxidative stress could be one of the earliest detectable events in the pathogenesis of AD—preceding the extracellular deposition of A*β* peptide and the formation of senile plaques and neurofibrillary tangles [16, 47].

Olfactory dysfunction is a common feature of AD affecting approximately 90% of AD cases [48]. Olfactory dysfunction results from the extensive cell loss and neurofibrillary tangles formation associated with AD that precede A*β* peptide deposition and occur in the olfactory bulb and olfactory centers (i.e., anterior olfactory nucleolus, periamygdaloid cortex, and anterior amygdala) in the early stages of AD [49, 50]. Evidence of oxidative stress has been observed in the olfactory epithelium of AD cases. Lipid peroxidation in the nuclear and cell membrane of olfactory neurons and epithelial cells of AD cases but not in age-matched normal neuroepithelial cells has been found [51, 52]. In addition, A*β* peptide deposition and paired helical filaments of tau protein (precursors of neurofibrillary tangles) are also substantially more frequent and more abundant in the olfactory epithelium of AD cases than in controls [48, 53]. Additionally, epidemiological studies indicate that olfactory dysfunction predicts an increased risk of cognitive decline with advanced age and takes place before the clinical manifestations of AD [50, 53, 54]. The occurrence of olfactory pathology in the early stages of AD and the identification of the nose as the portal of entry of airborne xenobiotics into the brain suggests AD pathology could be mediated by environmental agents such as air pollutants that could reach the brain through the olfactory epithelium [50, 55, 56].

## 3. The Role of Air Pollution in Alzheimer's Disease

### 3.1. Air Pollution

Atmospheric air pollution can be defined as the introduction of any chemical, physical, or biological pollutant—in the indoor or outdoor air—that modifies the natural characteristics of the atmosphere and harms human health and welfare [57]. Air pollutants can be released into the atmosphere from both natural (e.g., windblown dust, volcanoes, and wildfires) and anthropogenic sources (e.g., power plants, industries, and transportation). However, manmade sources are identified as the major contributor to indoors and outdoors air pollution [58].

 In the USA, NAAQSs for six principal air pollutants (i.e., O_3_, PM, CO, NO_x_, SO_2_, and lead) have been established to protect vulnerable populations (children, older adults, and individuals living with chronic diseases) against air pollutants toxicity. Primary and secondary NAAQS for these six criteria air pollutants are summarized in Table 2 [59].

### 3.2. Routes of Exposure

Despite the improvements made in ambient air quality, air pollution continues to be a prevalent environmental hazard in urban and rural areas. It is estimated that in the USA 146 million people live in areas that exceed the recommended air quality standards for at least one criteria air pollutant—in most cases, O_3_, PM, or both [13]. Human activities affect the timing, location, and degree of personal exposure to pollutants [60]. Adults, who generally spend most of their time inside (e.g., at home, workplace, and/or automobile), are more likely to be exposed to indoor air pollution while children, who spend more time than adults outside, are more likely to encounter outdoor air pollution [61].

Exposure to air pollution can occur through multiple routes. The release of pollutants into the atmosphere exposes humans to hazardous substances primarily by direct inhalation. Transport and deposition of air pollutants into water and soil also exposes humans to air pollutants through the ingestion of contaminated water and food [62]. Although representing a minor route of exposure, dermal contact with contaminated soil, dust, or water can also contribute to an individual's air pollutant intake [58, 62].

### 3.3. Air Pollution and Human Health

 Short- and long-term exposure to air pollution has consistently been linked to adverse health outcomes. Exposure to PM and O_3_ is associated with an increased cardiovascular and respiratory morbidity, mortality, and disability risk [63–68]. Recent estimates indicated that globally about 3% of adult cardiopulmonary disease mortality, 5% of cancers of the respiratory system mortality, and about 1% of acute respiratory infection mortality in children in urban areas are attributable to PM, which represents about one million premature deaths and 6.4 million years of life lost [64].

Epidemiologic studies have shown a positive association between cardiovascular hospitalizations and ambient NO_2_, CO, and PM levels [63, 65, 67, 68]. Exposure to air pollution has also been associated with reduced lung capacity in healthy individuals and increased risk of exacerbations, hospitalizations, and mortality in subjects with respiratory chronic diseases such as asthma and chronic obstructive pulmonary disease [66].

### 3.4. Air Pollution and Oxidative Stress

 Oxidative stress occurs when the production of ROS exceeds the natural antioxidant systems. This imbalance can result from exposure to pro-oxidant substances—ROS—present as air pollutants in the atmosphere [26, 57, 69]. The oxidative potential of air pollution relies on particle composition and size distribution and on the presence of transition metals and semivolatile and volatile organic chemicals [69]. Air pollutants can act directly as prooxidants of lipids and proteins or as free radical generators by promoting oxidative stress and inducing inflammatory responses after a threshold—a level at which natural antioxidant mechanisms are overwhelmed—is reached [58, 69, 70]. Autopsy samples from the frontal cortex of individuals exposed to urban air pollution indicates that air pollution produces frontal differential regulation of genes involved in oxidative stress, inflammation, cell proliferation, differentiation and apoptotic death, DNA damage, presynaptic signaling, membrane trafficking, and microtubule assembly and stability [71]. Once natural defense mechanisms are overwhelmed proinflammatory effects follow via the activation of redox-sensitive transcription factors such as NF*κ*B—nuclear factor kappa-light chain-enhancer of activated B cells [69]. Thus, given the large number of people exposed to air pollution, air pollutants could very well represent a prevalent source of environmentally induced ROS production [72] and thus a risk factor for AD—a neurodegenerative disorder mediated by oxidative stress.

### 3.5. Air Pollution and Alzheimer's Disease

 Evidence from toxicological studies using animal and cellular models indicates that individuals exposed to high levels of air pollution show damage in the olfactory mucosa, olfactory bulb, and frontal cortex region tissues—all similar to that observed in the AD brains [50, 71, 72]. The presence of neocortical hyperphosphorylated tau with pretangle material and amyloid-*β* diffuse plaques in the frontal cortex of individuals exposed to urban air pollution suggests a link between oxidative stress, neuroinflammation, neurodegeneration, and chronic exposure to high concentrations of air pollution [71]. Air pollution can also accelerate amyloid-beta-42 (A*β*-42) accumulation, which is a known cause of the neuronal dysfunction that precedes the formation of A*β* peptide plaques and neurofibrillary tangles [55, 73, 74]. In addition, human studies have shown that exposure to air pollution impairs cognitive function [75] and induces cerebrovascular damage [76].

**Figure 1:**
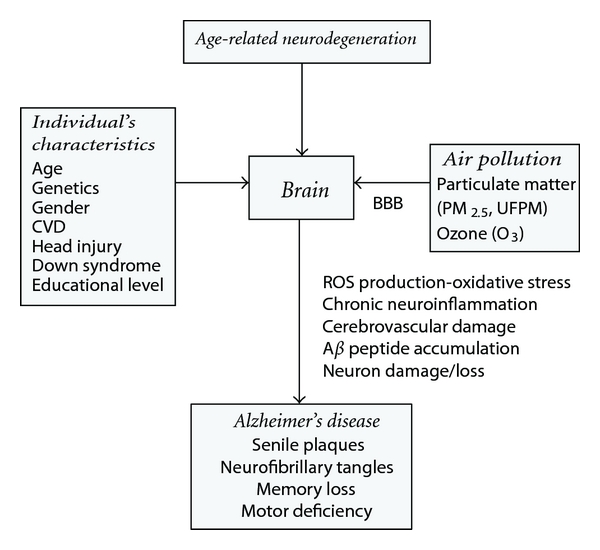
Interacting factors in the causality of Alzheimer's disease [1, 4–11, 17, 19, 28, 35, 72, 73]. Alzheimer's disease is the result of the interaction of aging, genetic predisposition, and environmental exposures such as air pollution in the etiology and pathogenesis of the disease. Air pollution is a prevalent environmental source of ROS that impacts the brain through the multiple pathways accelerating the development and clinical manifestation of Alzheimer's disease. BBB: blood-brain barrier; ROS: reactive oxygen species; CVD: cardiovascular disease.

Together, these findings support a plausible association between air pollution and AD. Also, they allow us to identify the human nose as the portal of entry of air pollutants into the brain [55, 56]. Although AD causality is multifactorial and thus the result of the interaction of several factors rather than of a single identifiable cause, there is enough evidence to identify air pollution as an important contributing factor to the development and expression of the disease. The interaction between aging, genetic predisposition, and air pollution in the causation of AD is depicted in Figure 1 [1, 4–11, 17, 19, 28, 35, 72, 73].

#### 3.5.1. Oxidative Mechanisms of Particulate-Induced Alzheimer's Disease Pathology

 Atmospheric particulate matter is a complex mixture of solid particles and liquid droplets commonly found in urban air, and it has shown to be associated with a variety of adverse health outcomes [58, 65, 77, 78]. The potential for PM to reach the central nervous system is directly associated with the particles size. Fine particles with an aerodynamic diameter of less than 2.5 *μ*m (PM_2.5_) and ultrafine particles of less than 0.1 *μ*m of aerodynamic diameter (UFPM) are the most significant for the pathogenesis of diseases of the central nervous system [72]. Ultrafine particles can reach the brain by transsynaptic transport after inhalation through the olfactory epithelium and uptake through the blood-brain barrier [16, 72, 74]. Deleterious effects of PM on the brain also vary depending of the number of particles, their chemical composition and physical characteristics, the amount of surface components that are translocate from the lung to other organs, and the velocity at which these particles and components are cleared from the system [72, 74].

The effects of PM on the brain are believed to be the result of two mechanisms. First, its ability to induce chronic respiratory and systemic inflammation by producing proinflammatory cytokines, which affect the blood-brain barrier, triggers neural-immune interactions and leads to chronic oxidative stress [16, 56, 75]. Second, its ability to directly produce ROS can damage the blood-brain barrier and increases the production of A*β* peptides [75]. Together these mechanisms are responsible for causing brain inflammation and accelerating the accumulation of A*β* peptide, both of which are associated with the neuronal dysfunction that precede the appearance of senile plaques and formation of neurofibrillary tangles [55, 74], which are the hallmarks of AD.

#### 3.5.2. Oxidative Mechanisms of Ozone-Induced Alzheimer's Disease Pathology

 Ozone is a gaseous air pollutant originated from photochemical reactions between NO_X_ and VOC in the troposphere. Ozone is the main component of smog and represents an important problem in urban areas, especially during the summer when sunlight is abundant. Emissions from industrial facilities, motor vehicle exhaust, and gasoline vapors are examples of important sources of NO_X_ and VOC—the precursors of O_3_.

Ozone is an ROS and powerful oxidizing agent [72, 79] capable of inducing oxidative stress state. Animal studies have indicated that the oxidative effects of O_3_ on the brain vary with the duration of the exposure [79] and show a dose-response relationship [36, 79]. This variation indicates that even though short O_3_ exposure induces ROS production, this occurs at a level that can still be compensated by antioxidant defense mechanisms. However, as the duration of the exposure increases, the production of ROS rises and finally reaches a threshold dose at which antioxidant defense mechanisms capacity is exceeded causing brain dysfunction [36]. This brain dysfunction is manifested as short- and long-term memory loss and motor deficiency in rats, all alterations that are positively related to the duration of O_3_ exposure [36, 79].

Besides causing motor deficiency and memory loss, O_3_ can also cause neuroinflammation, neuronal damage, and alterations of the cerebral vasculature [36, 72]. Moreover, O_3_-induced oxidative stress can cause dysregulation of inflammatory processes, progressive neurodegeneration, chronic loss to brain repair in the hippocampus, and brain plasticity changes in rats, which are comparable to those observed in AD disease patients [79]. Although there is evidence showing the oxidative changes caused by O_3_ on the brain, the mechanisms through which this gas reaches and affects the brain are yet to be understood [36, 72] and should consequently motivate future research efforts.

## 4. Future Directions

In this paper we discussed the current evidence describing an association between exposure to air pollution and AD. Although evidence from toxicological studies using animal and cellular models is abundant, epidemiological evidence is limited. Thus, the potential link between air pollution and AD at the population level remains unclear. More research is needed to characterize the association between exposure to air pollution and AD and its implications for public health.

At the individual level, efforts should be oriented toward determining the routes through which each air pollutant reaches the brain, as well as the biological mechanisms through which they contribute to the development and clinical manifestation of AD. Specifically, the effects of PM and O_3_ on the brain have been the focus of many studies; however, exposure to VOC is yet to be described. Also, and because air pollution consists of a mixture of different air pollutants (i.e., particles, liquid droplets, and gases), further investigation on the potential additive or synergistic effects between these pollutants is imperative.

The identification of air pollution as a factor in the pathogenesis and etiology of AD on the population level could provide a strong basis for implementing novel public health initiatives that could prevent AD for reaching epidemic proportions. By controlling the environmental factors that contribute to the pathogenesis of AD, public health professionals could also effectively minimize the burden AD is projected to place on worldwide healthcare systems in the decades to come.

## 5. Conclusion

Air pollution has consistently been identified as a significant environmental hazard and its association with cardiovascular and respiratory disease is well established. Recent reports from toxicological studies indicate the existence of an association between air pollution and central nervous system disease. Depending on their characteristics air toxicants can reach the brain through several pathways. The effects of air pollution on the brain then manifest as neuroinflammation, oxidative stress, and neurodegeneration.

Although AD causality is multifactorial, air pollution could increase an individual's risk of developing AD by accelerating age-related oxidative changes observed in the brain and hence represent a significant public health hazard. Therefore, the control of environmental factors such as air pollution could be a key factor in limiting the predicted increase in AD cases, as well as the burden it is expected to have on healthcare systems, worldwide.

Despite the many studies investigating the association between air pollution and AD, the role of air pollution in the causation and pathogenesis of this neurodegenerative disorder is not fully understood. Individual factors that could mediate the association between air pollution and AD such as age, nostril size, daily activities, and concomitant health conditions need further investigation. In addition, epidemiological studies looking at the association between air pollution and AD are few. Therefore, the implications of the association between air pollution and AD at a population level remain unclear. The predicted burden of AD on public health and the health care system should further motivate future research oriented toward providing evidence to obtain a better understanding of this association and guide preventive efforts.

## Figures and Tables

**Table 1:** Reactive Oxygen Species (ROS), Sources, and Defense Mechanisms [17–19, 22, 26].

ROS	
Superoxide (O_2_^•−^) Hydrogen peroxide (H_2_O_2_) Hydroxyl radical (^•^OH) Peroxyl radicals (ROO^•^) Alkoxyl radicals (RO^•^) Organic hydroperoxides (ROOR') Hypochlorous acid (HOCl) Peroxynitrite (ONOO−) Singlet oxygen (^1^O_2_)	
ROS sources	
*Endogenous * Mitochondria (byproducts of electron transport) oxidases (Oxidase-catalyzed reactions) Inflammation (neutrophils, macrophages) Cytochrome P450 reactions Arginine metabolism Peroxisomal fatty acid metabolism (peroxisomes, lipoxygenases) *Exogenous * Ionizing radiation (X–, *γ*−, UV) Chemotherapeutics Inflammatory cytokines (macrophages, neutrophils) Environmental toxins	
Antioxidant defense mechanisms	
*Enzymatic systems * Catalase (CAT) Superoxide dismutase (SOD) Glutathione peroxidase (GPx) *Nonenzymatic systems * DNA repair mechanisms Glutathione Vitamins (A,C, and E) Carotenes	

**Table 2:** National ambient air quility standards (NAAQS) for criteria air pollutants [59].

Criteria air pollutant	Primary standard		Secondary standard	
	Level	Averaging Time	Level	Averaging Time
(1) Ozone	0.075 ppm (2008 std)	8 hours	Same as primary	
(2) Particulate Matter			Same as primary	
PM_2.5_	15.0 *μ*g/m^3^	Annual		
	35 *μ*g/m^3^	24 hours	Same as primary	
PM_10_	150 *μ*g/m^3^	24 hours		
(3) Carbon Monoxide	9 ppm (10 mg/m^3^)	8 hours	None	
	35 ppm (40 mg/m^3^)	1 hour		
(4) Nitrogen Dioxide*	53 ppb	Annual	Same as primary	
	100 ppb	1 hour	None	
(5) Sulfur Dioxide	75 ppb	1 hour	0.5 ppm	3 hour
(6) Lead	0.15 *μ*g/m^3^	Rolling 3 month average	Same as primary	

*Although NAAQS cover the entire group of NO_X_, NO_2_ is use as indicator for this group.
